# Propofol attenuated TNF-α-modulated occludin expression by inhibiting Hif-1α/ VEGF/ VEGFR-2/ ERK signaling pathway in hCMEC/D3 cells

**DOI:** 10.1186/s12871-019-0788-5

**Published:** 2019-07-09

**Authors:** Yue Zhang, Xiaowei Ding, Changhong Miao, Jiawei Chen

**Affiliations:** 1 Department of Anesthesiology, Fudan University Shanghai Cancer Center, Shanghai, 200032 China; 2 Department of Oncology, Shanghai Medical College, Fudan University, Shanghai, 200032 China

**Keywords:** Occludin, TNF-α, Propofol, hCMEC/D3 cells

## Abstract

**Background:**

The levels of tight junction proteins (TJs), especially occludin, correlate with blood-brain barrier (BBB) disruption caused by inflammation in central nervous system (CNS). It has been reported that propofol, the most commonly used anesthetic, could inhibit inflammation response in CNS. In this study, we investigated the effects of tumor necrosis factor-α (TNF-α) and propofol on occludin expression in human cerebral microvascular endothelial cell line, D3 clone (hCMEC/D3 cells), and explored the underlying mechanisms.

**Methods:**

The hCMEC/D3 cells were treated with propofol, followed by TNF-α. The expression and phosphorylation of Hif-1α, VEGF, VEGFR-2, ERK, p38MAPK and occludin were measured by Western blot analysis. The cell viability of hCMEC/D3 cells was measured by cell counting kit-8.

**Results:**

TNF-α (10 ng/ml, 4 h) significantly decreased the expression of occludin, which was attenuated by propofol (25 μM). TNF-α induced Hif-1α/VEGF/VEGFR-2/ERK signaling pathway, while propofol could inhibit it. TNF-α induced the phosphorylation of p38MAPK, while propofol had no effect on it. In addition, the inhibitors of Hif-1α, VEGFR-2, and ERK could reduce the effect of TNF-α on occludin expression.

**Conclusion:**

TNF-α could decrease the expression of occludin via activating Hif-1α/ VEGF/ VEGFR-2/ ERK signaling pathway, which was attenuated by propofol.

**Electronic supplementary material:**

The online version of this article (10.1186/s12871-019-0788-5) contains supplementary material, which is available to authorized users.

## Background

Blood-brain barrier (BBB) is a multicellular structure and mainly consists of microvascular endothelial cells, astrocytes, pericytes, intercellular tight junctions and basal lamina [1]. It is accepted that BBB can protect central nervous system (CNS) from harmful molecules and pathogens in the peripheral blood circulation. A dysfunctional or impaired BBB allows augmented permeation of macrophages, leukocytes, endotoxins and bacteria into the CNS. Studies have revealed that the disruption of BBB may be involved in neuropathological status, such as postoperative cognitive dysfunction [2], Alzheimer disease [3], and dementia [4].

Cerebral microvascular endothelial cells, one of the most important components of BBB, are linked together through multiple tight junction proteins including claudins, occludin and zonula occludens (ZOs) [5]. The tight junctions play a crucial role in the biological functions of BBB. It has been reported that the decreased expression of tight junction proteins, especially occludin, correlates with the disruption of BBB [6, 7]. A study also revealed that truncation of occludin decreased transendothelial electrical resistance, suggesting a key role of occludin in the barrier function of BBB [8].

It has been reported that BBB disruption could be induced by many factors, including neuroinflammation, oxidative stress, and hypoxia [9–11]. Recent studies showed that inflammatory reactions may be the primary cause for BBB disruption [12]. Tumor necrosis factor-α (TNF-α) is one of the most important inflammation factors and plays an essential role in neuroinflammation. It has been reported that TNF-α could decrease the expression of occludin in endothelial cells [13]. However, the underlying mechanism of TNF-α-mediated occludin expression in microvascular endothelial cells has not been fully elucidated. Hypoxia inducible factor-1α (Hif-1α) has been reported to be activated by TNF-α and was correlated with occludin expression [14, 15]. However, the exact mechanism is unclear. As a direct target of Hif-1α, vascular endothelial growth factor (VEGF) can regulate paracellular permeability via the activation of extracellular regulated protein kinases (ERK) [16]. Therefore, we sought to investigate and verify these molecules in TNF-α-mediated occludin expression.

Propofol, a widely used intravenous anesthetic agent, has been reported to own anti-inflammation effects. Studies have revealed that propofol could protect hypoxia-induced inflammation in BV2 microglial cells [17]. It was also demonstrated that propofol inhibited sevoflurane-induced inflammation in human neuroglioma cells [18]. In this study, we sought to explore whether propofol could attenuate the effect of TNF-α on occludin expression and identify the underlying mechanisms.

## Materials and methods

### Cell culture and regents

Human cerebral microvascular endothelial cell (hCMEC) line, D3 clone, was purchased from Shanghai GuanDao biological engineering company and maintained in Dulbecco’s modified Eagle’s medium (DMEM; Hyclone, Australia); all culture media were supplemented with 10% fetal bovine serum (Gibco, Australia), 100 mg/mL streptomycin, and100 U/mL penicillin. The hCMEC/D3 cells were cultured in a humidified 5% CO_2_ atmosphere at 37 °C. The cells were passaged and cultured on reaching 80% confluence and the cell medium was changed every other day.

TNF-α powder was obtained from PeproTech China and dissolved in serum-free DMEM. Propofol (Sigma, St. Louis, MO, USA) was dissolved in dimethyl sulfoxide (DMSO; Sigma-Aldrich). The ERK inhibitor LY3214996 was purchased from Sigma Aldrich. SU5416, an inhibitor of VEGFR-2, and KC7F2, an inhibitor of Hif-1α, were purchased from MedChemExpress (Shanghai, China). KC7F2, SU5416, LY3214996 were dissolved in DMSO, respectively. The final concentration of DMSO was adjusted to 0.01% for each solution to avoid possible non-specific effects.

### Western blot analysis

After designed treatments, the cell extracts were collected and lysed with RIPA lysis buffer (Beyotime Biotechnology, Suzhou, China) supplemented with protease and phosphatase inhibitors (Roche, Rotkreuz, Switzerland). The lysates were mixed with 5× loading buffer and boiled for 5 min at 100 °C. Equivalent amounts of protein in each sample were separated by 10% or 12% SDS-polyacrylamide gel and transferred to a polyvinylidene fluoride membrane (Millipore, Billerica, MA) using a semidry electroblotting system. The membranes were blocked with 5% skim milk in phosphate-buffered saline-Tween 20 (PBST) for 1 h at room temperature, and then incubation with approprite primary antibody at dilution of 1:1000 at 4 °C overnight. The primary antibodies included: antibodies against occludin (Proteintech, Wuhan, China), p-ERK (CST, Massachusetts, USA), ERK (CST, Massachusetts, USA), p-p38MAPK(CST, Massachusetts, USA), p38MAPK(CST, Massachusetts, USA), p-VEGFR-2(CST, Massachusetts, USA), VEGF (CST, Massachusetts, USA), Hif-1α(CST, Massachusetts, USA) (Abcam, Shanghai, China), β-actin (Proteintech, Wuhan, China). After that, the membranes were washed with PBST and incubated with secondary antibodies conjugated with horseradish peroxidase (HRP) for 1 h at room temperature. After washing, the signals were detected using a LAS-4000 mini CCD camera (GE Healthcare). β-actin was used as internal control and the intensity of each protein band was normalized with that of β-actin. Each assay was performed in five replicates.

### Cell viability assay

The cell viability of hCMEC/D3 cells was measured by cell counting kit-8 (CCK8) (Beyotime Institute of Biotechnology, Shanghai, China). In this study, the hCMEC/D3 cells were seeded on 96-multiwell plates at a density of 5 × 10^3^ cells/well and cultured for 24 h. After designed treatment, the culture medium was replaced by 20 ml CCK8 along with 180 ml fresh culture medium and incubated in 37 °C for 2 h. The absorbance at a 450 nm wavelength was measured by a microplate reader (Synergy H4, Bio-Tek) and the cell viability curve was determined by calculating the mean value and standard deviation (SD) of the optical density for every 6 wells.

### Statistical analysis

Data were expressed as mean ± SD, and results were obtained from at least 5 separately performed experiments. Differences between groups were determined by one-way analysis of variance (ANOVA) followed by the Newman–Keuls test. InStat statistical program (GraphPad Software, San Diego, CA, USA) was used for the statistical analyses. All results in this study were considered statistically significant at a value of *p* < 0.05.

## Results

### TNF-α decreased the expression of occludin in hCMEC/D3 cells

**Fig. 1:**
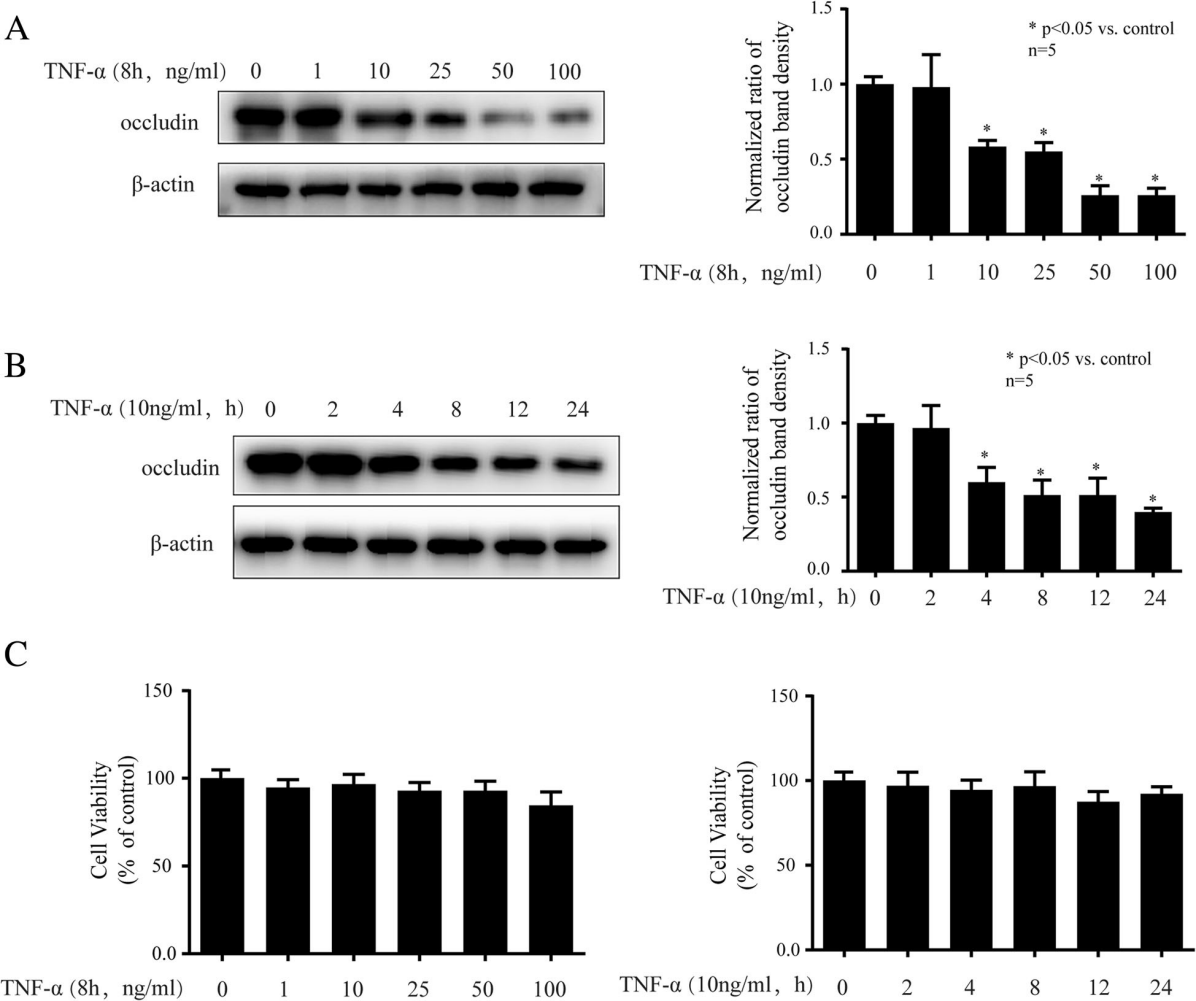
TNF-α could decrease the expression of occludin in hCMEC/D3 cells. **a** The hCMEC/D3 cells were treated with different concentrations of TNF-α (1, 10, 25, 50 and 100 ng/ml) for 8 h. Left side shows the image of a representative Western blot for occludin; right side is the plot of normalized ratios of optical densities. **b** The hCMEC/D3 cells were incubated with TNF-α (10 ng/ml) for different hours (2, 4, 8, 12, 24 h). Left side is the image of a representative Western blot for occludin; right side is the plot of normalized ratios of optical densities. **c** The cell viability of hCMEC/D3 cells after TNF-α treatment. β-actin was served as internal loading control. Data were represented as the mean ± SD. *p < 0.05, compared with the control group. Each assay was performed in five replicates

As shown in Fig. 1a, we treated different concentrations of TNF-α (1, 10, 25, 50, 100 ng/ml) for 8 h, and observed that 1 ng/ml TNF-α had no effect on occludin expression, while 10, 25, 50, 100 ng/ml TNF-α all significantly decreased occludin expression. It is noted that 10 ng/ml was the minimal dose of TNF-α to exert significant effect on occludin expression. Then we treated cells with 10 ng/ml TNF-α for different times (2, 4, 8, 12, 24 h), and found that 2 h treatment of TNF-α had no effect on occludin expression, while 4, 8, 12, 24 h treatment of TNF-α greatly decreased occludin expression (Fig. 1b). It is noted that 4 h was the minimal treatment time of TNF-α to exert significant effect on occludin expression. As shown in Fig. 1c, the cell viability was not changed by TNF-α at different concentrations (1, 10, 25, 50, 100 ng/ml) or different times (2, 4, 8, 12, 24 h). Then, we treated cells with 10 ng/ml TNF-α for 4 h in follow-up experiments to explore the underlying mechanism.

### Propofol inhibited the effect of TNF-α on occludin in hCMEC/D3 cells

**Fig. 2:**
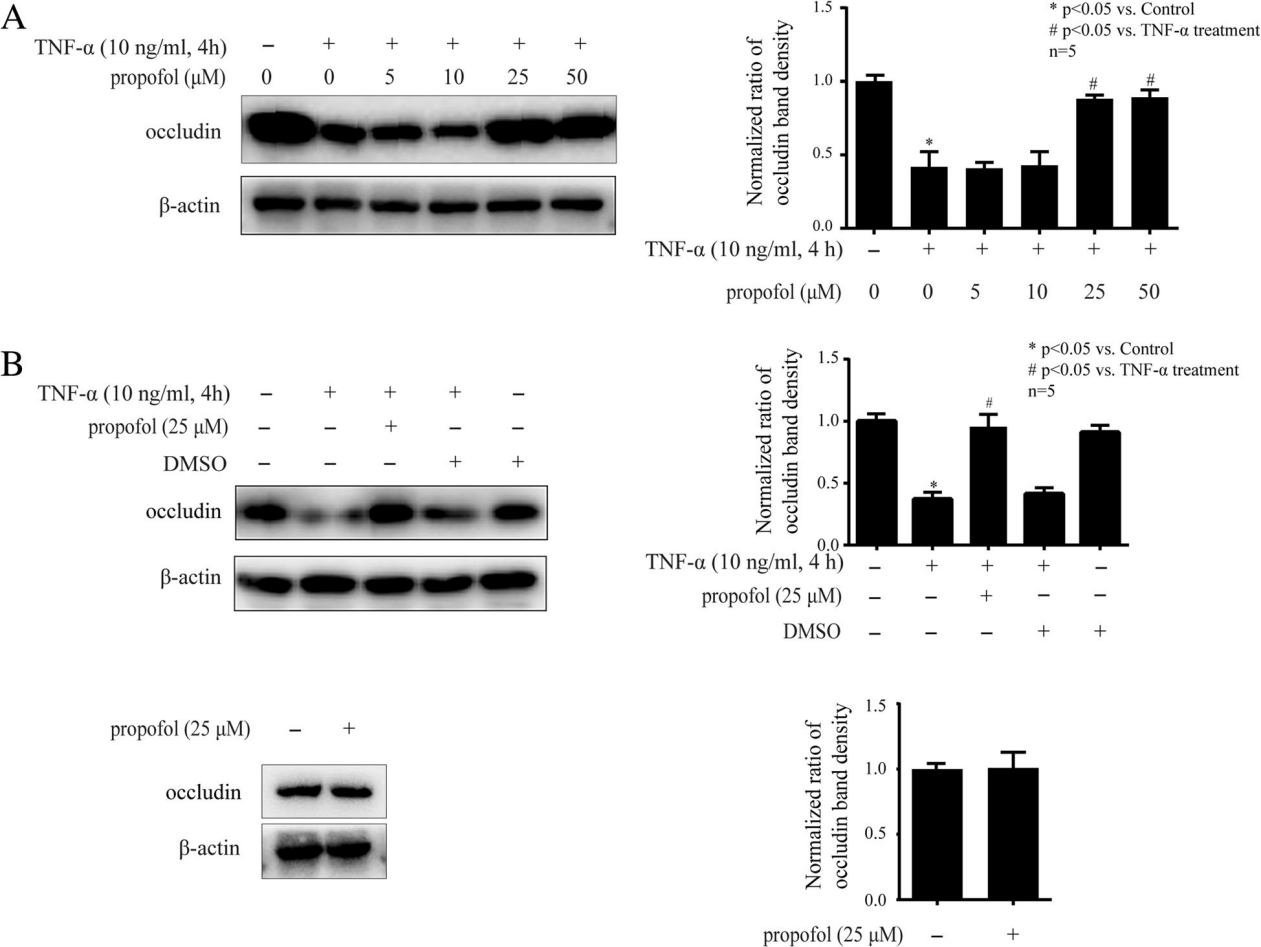
Propofol could inhibit TNF-α-modulated occludin expression in hCMEC/D3 cells. **a** The hCMEC/D3 cells were pretreated with different concentrations of propofol (5, 10, 25, 50 μM) for 2 h, followed by TNF-α treatment (10 ng/ml, 4 h). Left side is the image of a representative Western blot for occludin. Right side is the normalized ratios of optical densities. **b** DMSO served as solvent for propofol in this study. Left side is the protein expression of occludin in hCMEC/D3 cells that treated with TNF-α, propofol and DMSO (0.01%). Right side is the normalized ratios of optical densities. β-actin was served as internal loading control. Data were represented as the mean ± SD. *p < 0.05, compared with the control group. #*p* < 0.05, compared with TNF-α treatment group. Each assay was performed in five replicates

As shown in Fig. 2a, we pretreated hCMEC/D3 cells with different concentrations of propofol (5, 10, 25, 50 μM) for 2 h and followed by TNF-α treatment (10 ng/ml, 4 h). We found 5 or 10 μM propofol pretreatment had no effect on TNF-α-modulated occludin expression, while 25 and 50 μM propofol pretreatment significantly attenuated the TNF-α-modulated occludin expression. Please noted that 25 μM was the minimal dose of propofol to show significant effect. As shown in Fig. 2b, we detected that DMSO (0.01%), the solvent of propofol, had no effect on TNF-α-modulated occludin expression or basal occludin expression. In addition, we found that 25 μM propofol pretreatment alone had no effect on basal occludin expression. Therefore, 25 μM propofol pretreatment for 2 h was used in the following experiments to investigate the mechanisms of propofol.

### TNF-α decreased the expression of ZO-1, while propofol had no effect on it

As shown in Additional file 1: Figure S1, we treated hCMEC/D3 cells with different concentration of TNF-α (10, 50, 100 ng/ml) for 8 h, and observed that TNF-α had no effect on claudin-5 expression. In addition, we found that although TNF-α (10 ng, 4 h) decreased the expression of ZO-1, while 25 or 50 μM propofol could not repress the effect of TNF-α. Thus, we decided not to pursue further with claudin-5 and ZO-1 in this study.

### ERK was involved in the TNF-α and propofol-mediated occludin expression in hCMEC/D3 cells

**Fig. 3:**
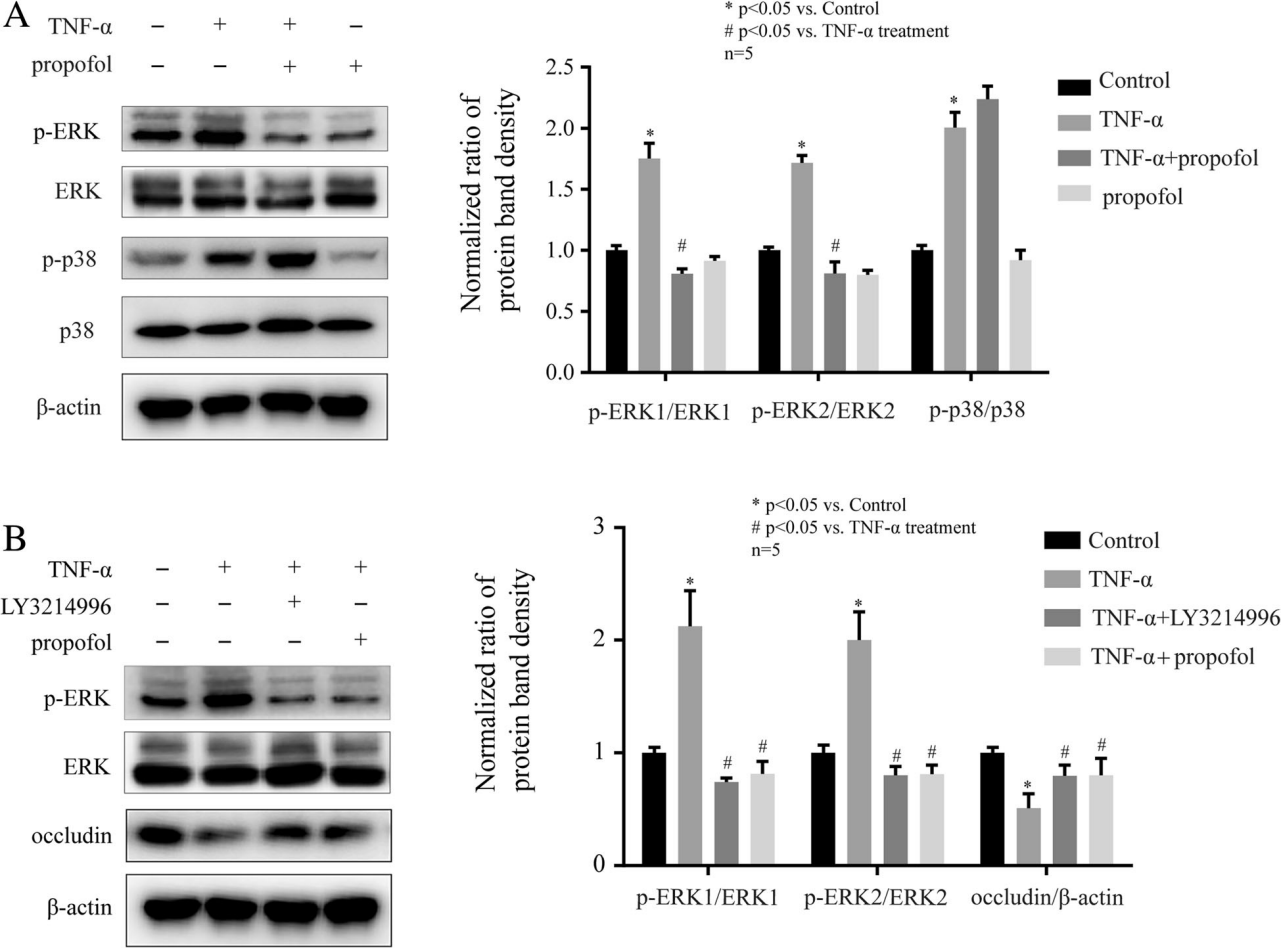
The phosphorylation of ERK was involved in the effects of propofol and TNF-α on occludin expression in hCMEC/D3 cells. **a** Left side is the protein expression of p-ERK, p-p38MAPK, ERK and p38MAPK in hCMEC/D3 cells that treated with TNF-α and propofol. Right side is the plot of normalized ratios of optical densities. **b** The hCMEC/D3 cells were treated with TNF-α, propofol, and ERK inhibitor (LY3214996, 5 μM). Left side is the protein expression of p-ERK, ERK, and occludin. Right side is the plot of normalized ratios of optical densities. β-actin was served as internal loading control. Data were represented as the mean ± SD. *p < 0.05, compared with the control group. #*p* < 0.05, compared with TNF-α treatment group. Each assay was performed in five replicates

It has been reported that the phosphorylation of ERK and p38MAPK may be involved in the expression of occludin [19]. As shown in Fig. 3a, TNF-α (10 ng, 4 h) induced the phosphorylation of ERK, which was attenuated by 25 μM propofol in hCMEC/D3 cells. TNF-α and propofol had no effect on the expression of ERK. Then, we found that the phosphorylation of p38MAPK was induced by TNF-α, while propofol had no effect on p38MAPK phosphorylation (Fig. 3a). To further confirm the role of ERK, we treated cells with the inhibitor of ERK. We found that 5 μM LY3214996, the inhibitor of ERK, significantly attenuated the effect of TNF-α on ERK phosphorylation and occludin expression. And these effects were similar to those of propofol (Fig. 3b).

### VEGF/ VEGFR-2 signaling pathway was involved in the effects of propofol and TNF-α on ERK and occludin in hCMEC/D3 cells

**Fig. 4:**
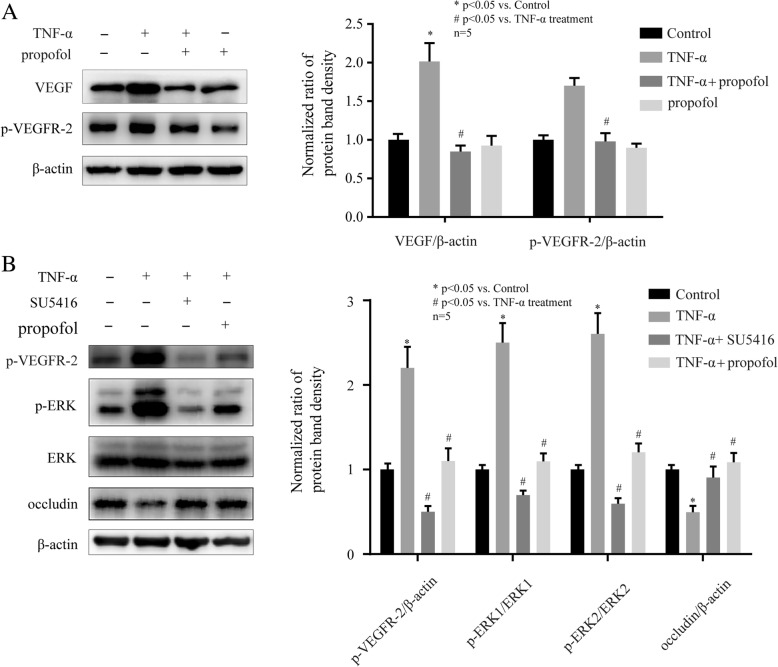
The VEGF/VEGFR-2 signaling pathway was involved in the effects of propofol and TNF-α on occludin expression in hCMEC/D3 cells. **a** Left side shows the image of a representative Western blot for VEGF and p-VEGFR-2 in hCMEC/D3 cells that treated with TNF-α and propofol. Right side is the plot of normalized ratios of optical densities. **b** The hCMEC/D3 cells were incubated with TNF-α, propofol, and VEGFR-2 inhibitor (SU5416, 10 μM). Left side shows the image of a representative Western blot for VEGF, p-VEGFR-2, p-ERK, ERK and occludin in hCMEC/D3 cells. Right side is the plot of normalized ratios of optical densities. β-actin was served as internal loading control. Data were represented as the mean ± SD. *p < 0.05, compared with the control group. #*p* < 0.05, compared with TNF-α treatment group. Each assay was performed in five replicates

It has been reported that VEGF could activate ERK pathway via the activation of VEGF receptor-2(VEGFR-2) [20]. As shown in Fig. 4a, TNF-α (10 ng, 4 h) induced the expression of VEGF and the phosphorylation of VEGFR-2 in hCMEC/D3 cells. Moreover, these effects were inhibited by 25 μM propofol. To further confirm the role of VEGF/VEGFR-2 signaling pathway, we treated cells with the inhibitor of VEGFR-2. We found that 10 μM SU5416, the inhibitor of VEGFR-2, markedly inhibited the effect of TNF-α on VEGFR-2 phosphorylation, ERK phosphorylation, and occludin expression in hCMEC/D3 cells. And these effects were similar to those of propofol (Fig. 4b).

### Hif-1α was involved in the effects of propofol and TNF-α on VEGF, VEGFR-2, ERK and occludin in hCMEC/D3 cells

**Fig. 5:**
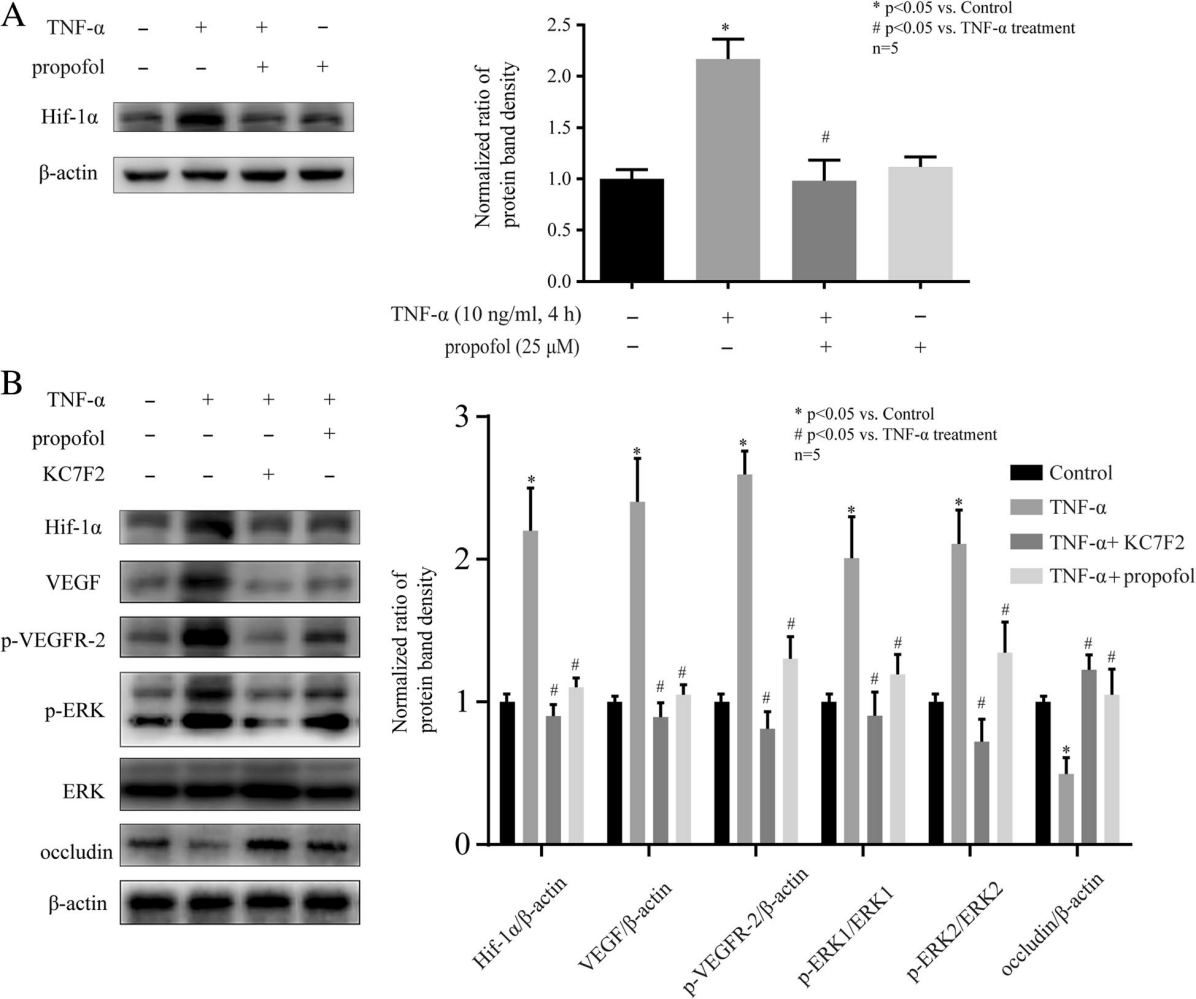
The expression of Hif-1α was involved in the effects of propofol and TNF-α on occludin expression in hCMEC/D3 cells. **a** Left side shows the image of a representative Western blot for Hif-1α in hCMEC/D3 cells that treated with TNF-α and propofol. Right side is the plot of normalized ratios of optical densities. **b** The hCMEC/D3 cells were treated with TNF-α, propofol, and Hif-1α inhibitor (KC7F2, 10 μM). Left side shows the image of a representative Western blot for Hif-1α, VEGF, p-VEGFR-2, p-ERK, ERK and occludin. Right side is the plot of normalized ratios of optical densities. β-actin was served as internal loading control. Data were represented as the mean ± SD. *p < 0.05, compared with the control group. #*p* < 0.05, compared with TNF-α treatment group. Each assay was performed in five replicates

It has been reported that VEGF could be regulated by Hif-1α [21]. As shown in Fig. 5a, TNF-α (10 ng, 4 h) induced the expression of Hif-1α, which was inhibited by 25 μM propofol. To further confirm the role of Hif-1α, we treated cells with the inhibitor of Hif-1α. We found that 10 μM KC7F2, the inhibitor of Hif-1α, significantly attenuated the effects of TNF-α on Hif-1α expression, VEGF expression, VEGFR-2 phosphorylation, ERK phosphorylation, and occludin expression in hCMEC/D3 cells. And these effects were similar to those of propofol (Fig. 5b).

### The confirmation of Hif-1α/ VEGF/ VEGFR-2/ ERK signaling pathway that induced by TNF-α

**Fig. 6:**
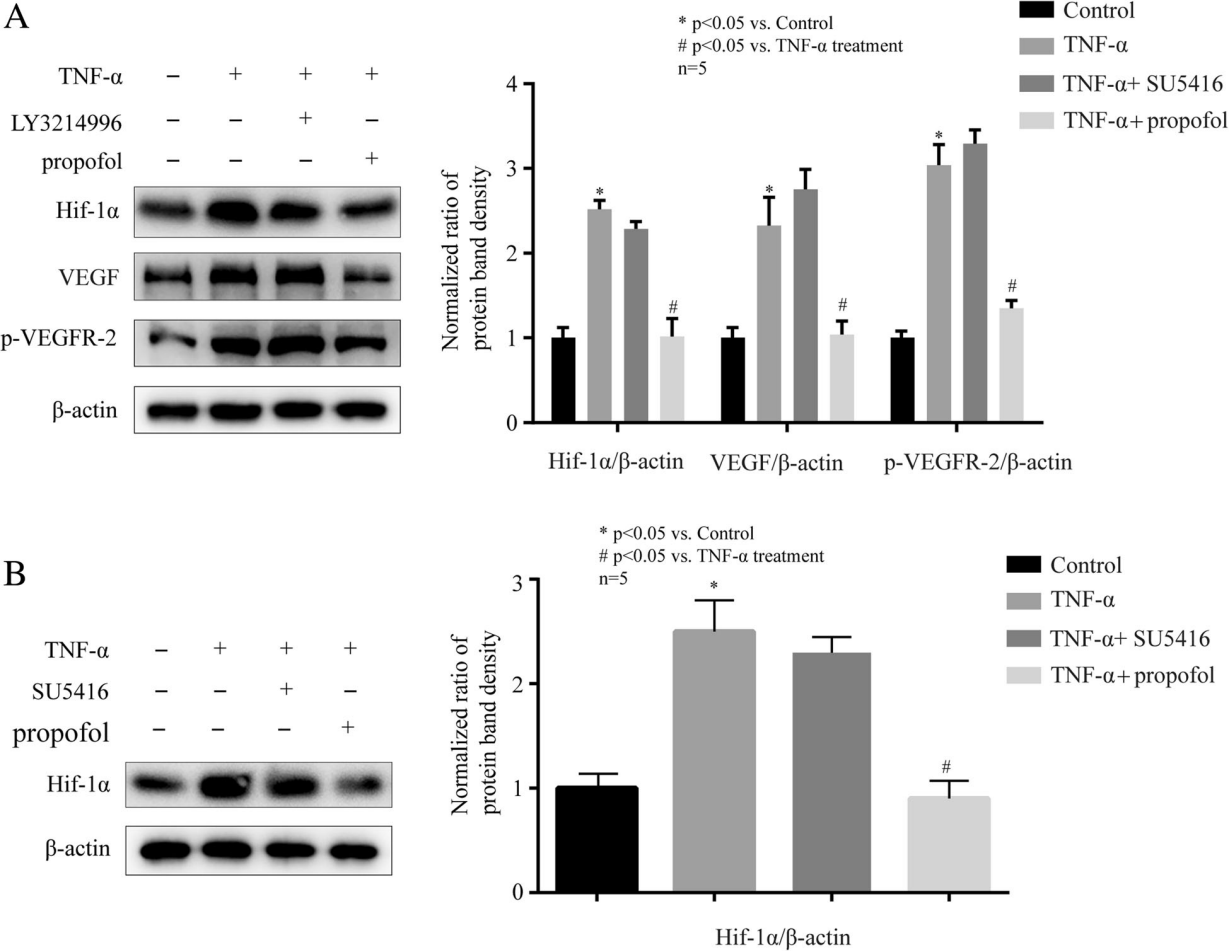
Propofol attenuated TNF-α-modulated occludin expression via inhibiting Hif-1α/ VEGF/ VEGFR-2/ ERK signaling pathway in hCMEC/D3 cells. **a** Left side shows the image of a representative Western blot for Hif-1α, VEGF, p-VEGF in hCMEC/D3 cells that treated with TNF-α, ERK inhibitor (LY3214996, 5 μM) and propofol. Right side is the plot of normalized ratios of optical densities. **b** Left side shows the image of a representative Western blot for Hif-1α in hCMEC/D3 cells that treated with TNF-α, VEGFR-2 inhibitor (SU5416, 10 μM) and propofol. Right side is the plot of normalized ratios of optical densities. β-actin was served as internal loading control. Data were represented as the mean ± SD. *p < 0.05, compared with the control group. #*p* < 0.05, compared with TNF-α treatment group. Each assay was performed in five replicates

To confirmed the sequential activation of Hif-1α, VEGF, VEGFR-2 and ERK, we treated hCMEC/D3 cells with ERK inhibitor (LY3214996), TNF-α (10 ng, 4 h), and 25 μM propofol. We found that 5 μM LY3214996, the inhibitor of ERK, had no effect on TNF-α-induced Hif-1α expression, VEGF expression and VEGFR-2 phosphorylation (Fig. 6a). Then, we treated cells with VEGFR-2 inhibitor (SU5416), TNF-α (10 ng, 4 h), and 25 μM propofol. We found that 10 μM SU5416, the inhibitor of VEGFR-2, had no effect on TNF-α-induced Hif-1α expression (Fig. 6b). Accordingly, we inferred that TNF-α decreased occludin expression via sequentially activating Hif-1α/ VEGF/ VEGFR-2/ ERK signaling pathway in hCMEC/D3 cells.

## Discussion

In this study, we observed that propofol inhibited TNF-α-modulated occludin expression. More importantly, our data implied that the Hif-1α/ VEGF/ VEGFR-2/ ERK signaling pathway was involved in this process.

It is known that BBB plays a crucial role in maintaining a stable environment of the CNS. Brain microvascular endothelial cells, one of the major components of BBB, are linked by intercellular tight junction protein complexes. Tight junctions among endothelial cells limit BBB permeability [22]. The tight junction proteins mainly include claudin-5, occludin and ZO-1 [23]. It has been reported inflammation plays a key role in tight junction disruption and many inflammation factors can modulate the expression of tight junction proteins [24]. For example, TNF-α was shown to decrease occludin expression in glomerular endothelial cells of mice [25]. It has also been revealed that TNF-α induced rat pulmonary microvascular endothelial cells injury via downregulating the expression of ZO-1 [26]. A study revealed that TNF-α reduced occludin and ZO-1 expression in pulmonary microvascular endothelial cells [27]. Consistently, we found that TNF-α significantly decreased the expression of occludin and ZO-1 in hCMEC/D3 cells. Interestingly, TNF-α was shown to reduce claudin-5 expression in pulmonary microvascular endothelial cells [26, 27], while we found TNF-α had no effect on claudin-5 expression in hCEMC/D3 cells. We inferred that TNF-α may exert different effects on occludin expression in different organs. To rule out the possibility that TNF-α affected cell viability. We treated hCMEC/D3 cells with TNF-α at different concentrations (1, 10, 25, 50, 100 ng/ml) and different times (2, 4, 8, 12, 24 h). And we found the cell viability was not changed by TNF-α (Fig. 1c). Accordingly, we inferred that the TNF-α-modulated occludin expression was not due to its effect on cell viability.

Propofol has been reported to own anti-inflammatory effects. It was revealed that propofol reversed TNF-α-induced human vascular endothelial cells apoptosis [28]. It has also been reported that propofol exerted anti-inflammatory effects in rats [29]. In this study, propofol inhibited lipopolysaccharide-induced acute lung injury by inhibition of cluster of differentiation (CD) 14 and Toll-like receptor (TLR) 4 expression [29]. In recent years, the anti-inflammatory effects of propofol in CNS attract widespread concerns. For example, it has been reported that propofol inhibited TNF-α-induced apoptosis in mouse hippocampal HT22 cells [30]. A study also revealed that propofol inhibited the inflammation and apoptosis of mouse microglia BV2 cells [17]. Here, we wondered if propofol attenuated the effect of TNF-α on the expression of occludin and ZO-1. As shown in Fig. 2, we found that the effect of TNF-α on occludin expression was markedly attenuated by 25 μM propofol, which is within clinical plasma concentration (5-50 μM) during the induction and maintenance of general anesthesia [31]. In addition, the supplementary data showed that propofol had no effect on TNF-α-modulated ZO-1 expression. Although it has been reported that propofol attenuated hypoxia-modulated ZO-1 expression in mouse brain microvascular endothelial cells [27, 32]. We reasoned that hypoxia and inflammation may reduce ZO-1 expression through different intracellular signaling pathway, and that propofol may have different effect on these pathways.

Hif-1α has been reported to be involved in occludin expression [33]. In addition, the role of Hif-1α in tight junction damage has recently become evident [14]. Although Hif-1α was usually induced by hypoxia, studies showed many inflammation mediators induced the expression of Hif-1α [15, 34]. Consistently, we found TNF-α increased Hif-1α expression in hCMEC/D3 cells, while propofol repressed it. In addition, we found that the inhibitor of Hif-1α repressed TNF-α-modulated occludin expression as propofol, suggesting Hif-1α may play a key role in the effect of propofol and TNF-α on occludin expression.

VEGF is a homodimeric 45-kDa glycoprotein and is secreted by a variety of cells including cerebral microvascular endothelial cells [35]. It is accepted that VEGF can affect endothelial cell survival and function. It has been revealed that VEGF was involved in occludin expression [36]. The study revealed that the level of occludin was lowered by VEGF treatment and that VEGF increased BBB permeability in brain microvascular endothelial cells monolayer cultures [36]. It has also been reported that VEGF was induced by Hif-1α in endothelial progenitor cell [37]. VEGF exerted its physiological function via interacting with receptors on vascular endothelial cells [38]. The receptors of VEGF (VEGFR) are receptor tyrosine kinases, and include VEGFR-1, VEGFR-2, and VEGFR-3 [39]. Although these receptors are highly similar in amino acid sequence, different VEGFRs have disparate functions and play key role in different pathological processes and signal transductions [40]. Previous studies indicated that VEGFR-1 was mainly involved in angiogenesis [41] and VEGFR-3 was involved in migration and invasion in cancer cells [42], while VEGFR-2 was closely involved with inflammation response [43]. It has been reported that VEGF activated VEGFR-2 to promote its auto-phosphorylation to generate p-VEGFR-2 [44], which exerted specific effects on intercellular permeability [45]. As shown in Fig. 4a, the expression of VEGF and the phosphorylation of VEGFR-2 could be induced by TNF-α, while propofol attenuated the effects of TNF-α. Then, we found that the inhibitor of VEGFR-2 attenuated TNF-α-modulated occludin expression. Furthermore, our data also showed that the inhibitor of Hif-1α markedly decreased VEGF expression and VEGFR-2 phosphorylation as propofol. Our finding demonstrated that TNF-α modulated occludin expression via Hif-1α/VEGF/VEGFR-2 signaling pathway, which was also involved in the effect of propofol.

Studies have revealed that the breakdown of BBB was due to the activation of ERK signaling pathway [46]. As one of the downstream molecules of VEGF/VEGFR-2 signaling pathway [47], ERK has been reported to be involved in the expression of occludin [48]. In our study, we found that TNF-α induced the phosphorylation of ERK, which was attenuated by propofol. And we also demonstrated that the inhibitor of ERK significantly alleviated the effect of TNF-α on occludin expression. In addition, we found the phosphorylation of ERK was repressed by the inhibitor of Hif-1α and the inhibitor of VEGFR-2, suggesting the role of Hif-1α/ VEGF/ VEGFR-2/ ERK signaling pathway in propofol- and TNF-α-modulated occludin expression.

It has been reported that p38MAPK was activated by TNF-α and played a crucial role in occludin expression [13]. As shown in Fig. 3a, we found TNF-α induced the phosphorylation of p38MAPK, while propofol had no effect on it. In addition, the inhibitor of ERK almost completely blocked TNF-α-modulated occludin expression in hCMEC/D3 cells. Thus, we inferred that compared with ERK, p38MAPK plays a minor role in TNF-α-modulated occludin expression.

In conclusion, our data strongly implied that propofol exerts protective effect on TNF-α-modulated occludin expression via inhibiting Hif-1α/ VEGF/ VEGFR-2/ ERK signaling pathway. We have realized this needs to be further confirmed in the animal study. Actually, we are planning to examine the protective effect of propofol on TNF-α-modulated occludin expression and BBB impairment in the mouse model.

## Additional file

Additional file 1: **Figure S1.** (a) TNF-α had no effect on the expression of claudin-5 in hCMEC/D3 cells. The hCMEC/D3 cells were treated with different concentrations of TNF-α (10, 50 and 100 ng/ml) for 8 h. Left side shows the image of a representative Western blot for claudin-5; right side is the plot of normalized ratios of optical densities. (b) TNF-α could decrease the expression of ZO-1, while propofol could not attenuate it. Left side is the protein expression of ZO-1 in hCMEC/D3 cells that treated with TNF-α and propofol; right side is the plot of normalized ratios of optical densities. β-actin was served as internal loading control. Data was repressed as the mean ± SD. *p < 0.05, compared with the control group. Each assay was performed in five replicates. (ZIP 598 kb)

## Data Availability

The datasets used or analysed during the current study available from the corresponding author on reasonable request.
