# Supplementary figures and images for: Propofol attenuated TNF-α-modulated occludin expression by inhibiting Hif-1α/ VEGF/ VEGFR-2/ ERK signaling pathway in hCMEC/D3 cells
Source: BMC Anesthesiol. 2019 Jul 9;19:127. doi: 10.1186/s12871-019-0788-5 (PMC6617648; doi:10.1186/s12871-019-0788-5)

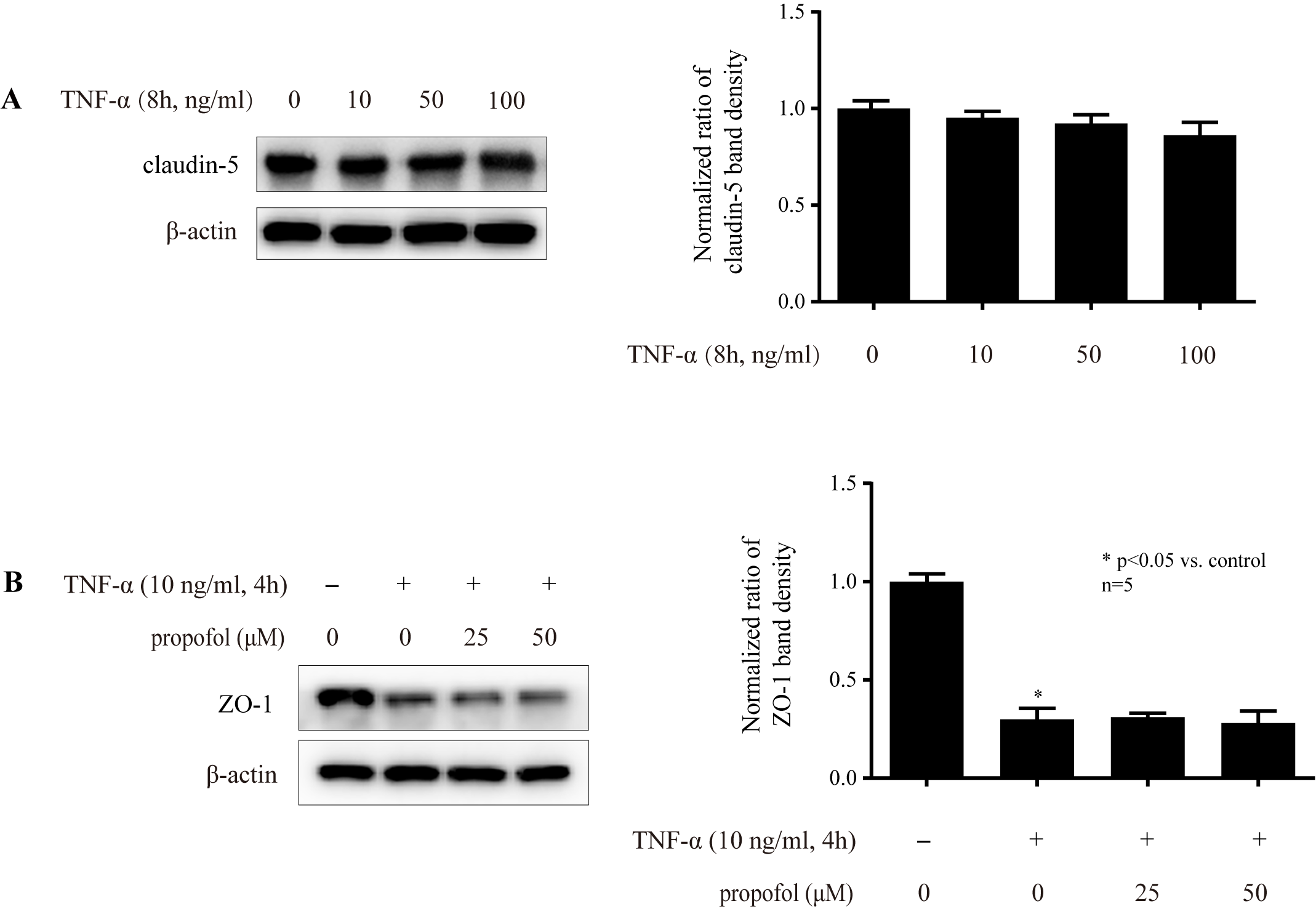

Supplement: Supplementary file 1 — Figure S1. (a) TNF-α had no effect on the expression of claudin-5 in hCMEC/D3 cells. The hCMEC/D3 cells were treated with different concentrations of TNF-α (10, 50 and 100 ng/ml) for 8 h. Left side shows the image of a representative Western blot for claudin-5; right side is the plot of normalized ratios of optical densities. (b) TNF-α could decrease the expression of ZO-1, while propofol could not attenuate it. Left side is the protein expression of ZO-1 in hCMEC/D3 cells that treated with TNF-α and propofol; right side is the plot of normalized ratios of optical densities. β-actin was served as internal loading control. Data was repressed as the mean ± SD. *p < 0.05, compared with the control group. Each assay was performed in five replicates. (ZIP 598 kb) [file 12871_2019_788_MOESM1_ESM.zip › supplementary materialR6.tif]
